# Muscle and Bone Implications of GLP‐1 Receptor Agonists and Fragility Fractures in Older Adults With Diabetes

**DOI:** 10.1002/jcsm.70371

**Published:** 2026-08-23

**Authors:** Joseph D. Abraham, Andrew J. S. Coats, Stefan D. Anker

**Affiliations:** ^1^ The Heart and Vascular Center and The Carl and Edyth Lindner Research Center The Christ Hospital Cincinnati Ohio USA; ^2^ Heart Research Institute Sydney New South Wales Australia; ^3^ Department of Cardiology (CVK), German Heart Center Charité, German Centre for Cardiovascular Research (DZHK) Partner Site Berlin Charité Universitätsmedizin Berlin Germany

Glucagon‐like peptide‐1 receptor agonists (GLP‐1RAs) have long treated type 2 diabetes mellitus (T2DM) and have exploded as a treatment for obesity and cardiovascular disease risk reduction in overweight and obese patients [1, 2, 3, 4, 5]. However, the risks associated with GLP‐1RAs are still being appropriately described, especially when obesity is seen in other chronic disorders such as heart failure [6, 7]. Specifically, unintentional effects on skeletal health and body composition need to be better studied. Kasher Meron et al. [8] provide important insight into these unintended consequences by examining the relationship between GLP‐1RA initiation and fragility fracture risk in older adults with T2DM.

Using a large population‐based cohort of over 46 000 individuals aged ≥ 65 years, the authors demonstrated that GLP‐1RA users, compared to alternative glucose‐lowering therapies, experienced a modest but statistically significant increase in fragility fractures (HR 1.11, 95% CI 1.01–1.21) [8]. The magnitude of this signal should not be dismissed. Older adults with T2DM already carry an elevated baseline fracture risk and this is especially prominent in women [9, 10, 11]. Therefore, even this relatively modest increase in risk may translate into meaningful harm. Importantly, the Meron et al. [8] analysis accounted for the competing risk of death already present in this population for the main outcome.

These findings contribute to a growing and previously somewhat conflicting body of literature regarding incretin‐based therapies and bone health. Some earlier studies have found mixed skeletal effects, including negative neutral or positive effects [12]. However, such studies were limited in duration, studied different populations and, in regard to weight‐loss, used less potent agents compared to contemporary ones such as semaglutide or tirzepatide. This current analysis is stronger by utilizing a national database instead of relying on meta‐analysis of studies that decide to publish their fracture risk data and includes the more currently common GLP‐1 RAs.

Importantly in the field of sarcopenia and cachexia, body composition versus total weight loss is becoming more important, especially with ageing populations and the increasing prevalence of chronic disease‐associated frailty [13, 14, 15]. GLP‐1RAs are not adipose‐selective. Recent preclinical work also suggests that intermittent GLP‐1RA exposure may worsen body‐composition trajectories, with treatment cycling associated with increased adiposity relative to lean mass and attenuated weight‐loss response after repeated exposure [16].

Clinical trials are underway to mitigate this adipose nonselectivity with combination drug use [17, 18]. Particularly in older adults, a population where sarcopenia is more prevalent, loss of skeletal muscle may be more detrimental [19]. Further, investigations into cancer cachexia and the molecular mechanisms may lead to more targeted treatments regarding body composition [20]. The ACT‐ONE trial has studied the use of s‐pindolol, an anabolic‐catabolic transfer agent, reporting preserved lean mass in specific cachexia patients [21]. Translating this, ongoing work is underway to study s‐pindolol in simultaneous and post‐GLP‐1RA therapy, including additional outcome measurements on bone composition [22].

One weakness of the study by Kasher Meron et al. [8] is that it does not include direct measures of bone mineral density, muscle mass or functional status. However, the large number of individuals and events do reinforce that the signal is real, but the magnitude may be affected by confounding. They do state in their findings that the GLP‐1RA group was associated with lower all‐cause mortality; however, a significant weakness of this finding was mortality was not stratified by the occurrence of fracture events. Future analysis should stratify mortality based off the presence of fractures, as fragility fractures are independently associated with increased mortality in older adults [23].

This study should not diminish the cardiometabolic benefits of GLP‐1RA use but should prompt a more integrated and multidisciplinary approach to their use. Baseline and longitudinal assessments may be necessary in selected patients, with possible interventions including improved nutrition or protein intake, resistance exercises or medication adjustments [24]. Bone density assessments may be required more often in high‐risk individuals.

Ultimately, the expanding study, and use, of GLP‐1RAs requires studies to look at both the benefits and negative effects of these drugs. This work highlights the potential skeletal effects in a high‐risk population. The results reflect a need to improve pharmacotherapy and is a present challenge currently being targeted to overcome. Jensen et al. [25] studied bone health (hip, spine and forearm) after diet‐induced weight loss followed by a 1‐year intervention with exercise, the GLP1 RA, liraglutide or both combined. They showed in 195 participants that compared with those taking liraglutide alone where BMD decreased at the hip (mean change, −0.013 g/cm^2^; 95% CI, −0.024 to −0.001 g/cm^2^; *p* = 0.03) and spine (mean change, −0.016 g/cm^2^; 95% CI, −0.032 to −0.001 g/cm^2^; *p* = 0.04), those undergoing exercise as well had no significant change in BMD. More prospective trials in larger numbers are needed to see how major these effects of GLP1RA's can be, how long they last and how they can be avoided if they prove to translate into significant fracture risk [26].

## Funding

The authors have nothing to report.

## Conflicts of Interest

The authors declare no conflicts of interest.

## Data Availability

Data sharing is not applicable to this article, as no datasets were generated or analysed during the current study.
